# Evaluation of the antibacterial efficacy of diode laser and zinc oxide nanoparticles as cavity disinfectants following partial caries removal: a randomized controlled clinical trial

**DOI:** 10.1038/s41405-025-00373-1

**Published:** 2025-12-16

**Authors:** Sarah Khattab, Rasha Raafat, Mona Riad

**Affiliations:** 1 Conservative Dentistry Department, Faculty of Dentistry, Al Ahram Canadian University, Cairo, Egypt; 2 Conservative Dentistry Department, Faculty of Dentistry, Cairo University, Cairo, Egypt; 3 Conservative Dentistry Department, Faculty of Dentistry, Cairo University, Cairo, Egypt

**Keywords:** Bonded restorations, Restorative dentistry

## Abstract

**Background:**

Dental caries remains one of the most common chronic conditions worldwide, often requiring treatment that balances effective bacterial control with preservation of healthy tooth structure. In recent years, researchers have explored novel antimicrobial approaches that can be used during minimally invasive caries treatments. Various adjunctive methods have been employed to inhibit the growth of residual bacteria in deep carious lesions. While chemical disinfectants have demonstrated notable antibacterial properties, their effectiveness can be compromised by some side effects, including adverse interactions with restorative materials and potential alterations to dental tissue structure. Consequently, there is a growing need for alternative antibacterial approaches. Among the latest advancements in cavity disinfection are diode lasers and zinc oxide nanoparticles.

**Aim:**

Considering the limited number of clinical studies investigating both agents as cavity disinfectants, the primary aim of the present clinical trial was to evaluate the antibacterial effectiveness of a diode laser (976 nm) and zinc oxide nanoparticles in an ethanol-based solution against cariogenic bacteria, specifically *Streptococcus mutans*, when applied as cavity disinfectants.

**Materials and methods:**

Thirty patients with deep cervical carious lesions were randomized into two groups (*n* = 15). After selective caries removal, baseline carious dentin samples were collected (control). One group received cavity disinfection using a 976 nm diode laser at 1.3 W output, while the other was treated with zinc oxide nanoparticles (50 μg/mL ethanol-based solution). Zinc oxide nanoparticles were prepared and characterized using transmission electron microscopy and X-ray diffraction to confirm morphology and crystallinity. Post-intervention dentin samples were collected via colony-forming units (CFU) for microbiological analysis.

**Results:**

Transmission electron microscopy revealed spherical zinc oxide nanoparticles with diameters averaging 18 nm. X-Ray diffraction confirmed high crystallinity. Both the laser diode and zinc oxide nanoparticles groups showed significant reductions in colony forming unit post-disinfection (*p* < 0.001). The diode laser group had significantly higher colony forming unit (mean: 8.27 ± 0.80 CFU/mL) compared to the zinc oxide nanoparticles group (mean: 3.47 ± 0.74 CFU/mL). Percentage of bacterial reduction was lower in the laser diode group (93.77%) than the ZnO NPs group (97.4%).

**Conclusion:**

Spherical-shaped zinc oxide nanoparticles with particle sizes around 25 nm and 50 μg/mL concentration in ethanol-based solution demonstrated superior antibacterial efficacy, suggesting they are a promising alternative for cavity disinfection in conservative caries management.

**Clinical significance:**

The results of the trial open new possibilities for improving patient outcomes by integrating more targeted and conservative disinfection options into everyday dental practice.

**Clinical trial registration:**

This study was registered on clinical trial (www.ClinicalTrials.gov).

## Introduction

Dental caries remains one of the most prevalent oral diseases globally. Streptococcus mutans, the primary etiological agent, contributes significantly to carious lesion formation [1]. The longevity of restorative treatments is challenged by residual cariogenic bacteria following caries removal [2]. To improve disinfection efficacy, adjunctive agents like lasers and nanoparticles have been introduced.

Clinical trials with long-term follow-ups demonstrated that Streptococcus mutans persists under restorations and have an important role in the development of secondary caries. Eventually, failure of the restoration and pulp involvement occurs. So, if the cavity is correctly sealed after removing all caries on the side walls, cariogenic bacteria are deprived of their nutrition and become inactivated [3]. Accordingly, using cavity disinfectants such as chlorhexidine (CHX), sodium hypochlorite, lasers, and nanoparticles has become more reasonable in clinical practice [4].

Management of carious lesions has been interestingly shifting towards a more conservative approach, with a better understanding of the caries process. The new approach aims at preserving the healthy tooth structure, thus prolonging tooth vitality. This can be achieved through various techniques, such as partial caries removal. This method is considered less invasive and preserves deeper layers of dentin, which are not bacterially contaminated and can re-mineralize to protect the pulp-dentin complex [5].

A promising candidate for cavity disinfection is the laser, which has been of valuable use in dentistry since it was first introduced by Maiman in 1960. Laser has played an effective role in enhancing the efficiency and comfort of treatment. Diode Lasers are the type of lasers produced by stimulation of Gallium and Arsenide, operating at a wavelength range of 810–980 nm [6]. They have various dental applications such as caries diagnosis and prevention, bleaching, cavity preparation, root canal disinfection, and treatment of dentin hypersensitivity [7]. The laser diode can produce an antibacterial effect on enamel, dentin, and carious tissues with minimum thermal damage on the remaining sound dental tissues [8]. The antimicrobial effect of the laser diode depends on its photothermal and photo-disruptive effects on the target cells [9]. Moreover, diode laser was found to produce effective antimicrobial activity when used in disinfection of root canals, as it can reach bacteria in the deepest layers of dentin, owing to its high water-transmission capacity. It was also found that at certain wavelengths (980 nm), the laser diode was able to induce changes in the surface of dentin which increases its bactericidal effect [10].

Nanomaterials are a family of materials with particle sizes ranging from 1 to 100 nm. This nano size provides unique physicochemical properties compared to bulk materials [11]. Zinc oxide nanoparticles are particularly promising due to their inherent antibacterial properties. These properties are attributed to the generation of reactive oxygen species (ROS), Zn2+ ion release. The released ROS interacts with bacterial cell membranes leading to damage of the bacterial cell [12]. ZnO NPs exhibit low cytotoxicity and high biocompatibility, which makes them suitable for dental applications. Their nanoscale size enables better penetration into dentinal tubules, enhancing antimicrobial activity. Applications in restorative dentistry include their incorporation into bonding agents, composites, and as stand-alone disinfectants [12].

Given the limited clinical trials assessing laser diode and ZnO NPs in this context, this study compares the antibacterial effects of both as disinfectants following partial caries removal.

Therefore, more clinical trials were needed to support the effectiveness of both methods. The proposed hypothesis was that there would be a difference between the bacterial count of S. mutans before and after cavity disinfection using laser diode and ZnO NPs in deep carious lesions.

## Materials and methods

### Laser diode

**Fig. 1:**
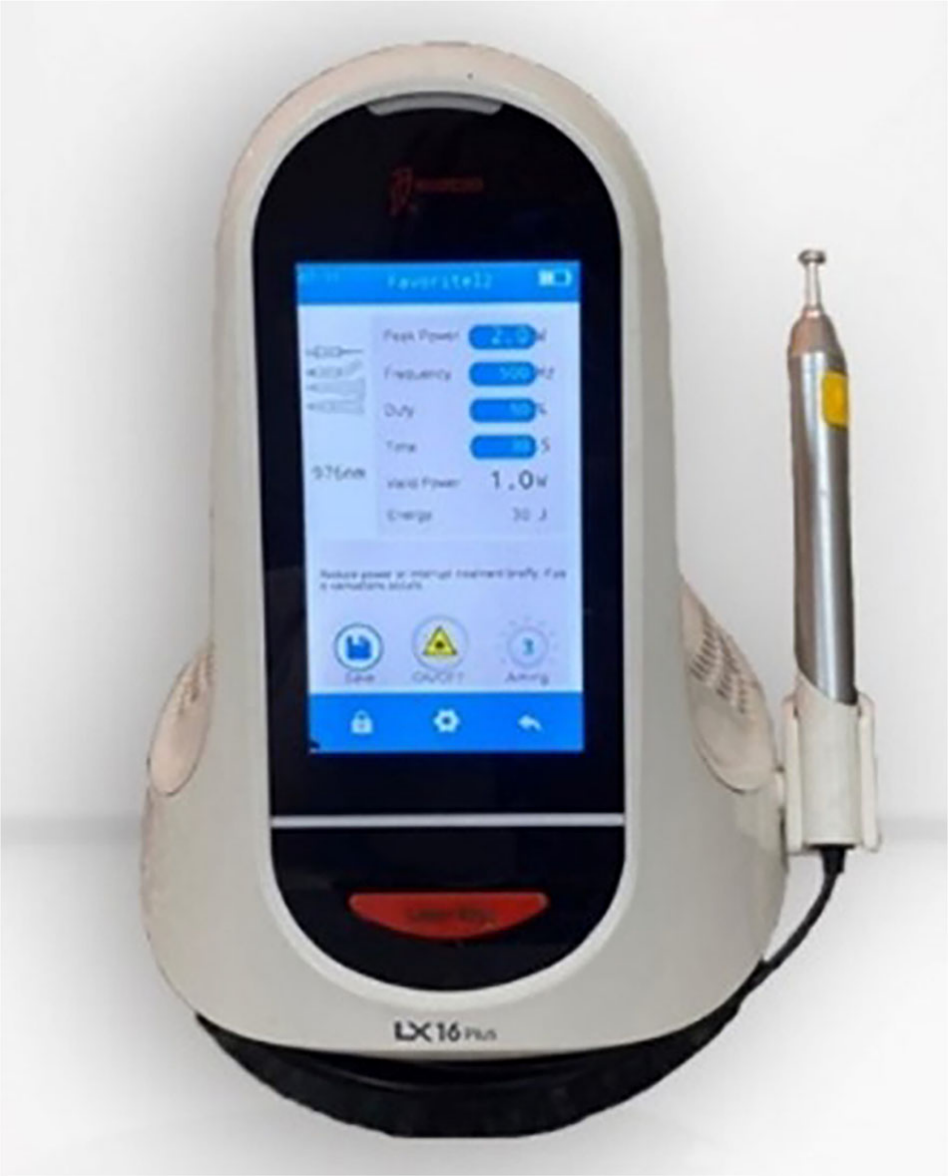
Laser diode device used in cavity disinfection. The diode laser was operated at wavelength 976 nm at an output power 1.3 W, in a continuous mode.

The laser light was transferred using a diode laser device (Woodpecker LX16 plus, made in China) (Fig. 1), through a flexible fiber optic tip (400 µm) attached to a special handpiece. The output power was adjusted at 1.3 W, and the wavelength at 976 nm [9].

### Preparation of zinc oxide nanoparticles

Zinc oxide nanoparticles (ZnO-NPs) were synthesized under mild, straightforward conditions at 60 °C without organic additives and without additional calcination. Briefly, zinc acetate dihydrate (0.24 mol) was dissolved in methanol, after which a methanolic NaOH solution was added dropwise at 60 °C under constant stirring for at least 1 h. The reaction mixture turned white and turbid, indicating nanoparticle formation. The precipitate was filtered, repeatedly washed with methanol to remove unreacted salts, and dried at 100 °C. For clinical application, 1 mg of ZnO-NPs powder was dispersed in 20 mL of absolute ethanol (99.9%, Alamia, Egypt) to yield a final concentration of 50 µg/mL (0.005% w/v). The suspension was freshly prepared before each application, sonicated for 13–15 min to minimize agglomeration, and handled under aseptic conditions using sterile graduated tubes and disinfected pipette tips to maintain sterility [13].

Particle size distribution and zeta potential were not measured in the present trial due to resource limitations; however, the methanolic synthesis route employed typically yields ZnO NPs in the 20–100 nm range, with D50 values around 50–60 nm and zeta potential between +20 and +30 mV, parameters consistently associated with effective antibacterial activity [13–16].

### Characterization of zinc oxide nanoparticles

The morphology of the synthesized ZnO NPs, including the size, shape, and uniformity, was detected using high-resolution transmission electron microscope (HR-TEM) (JEOL, model: JEM-2010).

fixed at an accelerating voltage of 200 kV, and fitted with a Gatan Digital Camera (Model: Erlangshen ES500). The X-ray diffraction (XRD) method was employed in this study to analyze the crystallinity and phase structures of zinc oxide nanoparticles (ZnO NPs). This was done using an XRD 6100 diffractometer with CuKa1 radiation (*k* = 1.54056 Å) over a 2*θ* range of 10–80°, with an operating voltage of 40 kV and a current of 30 mA. The potential of the nanoparticles was assessed by measuring the zeta potential at 25 °C using a Malvern Zetasizer model Nano ZS-90 [14].

### Study design

This comparative, randomized clinical trial was carried out on adult patients at the Department of Conservative Dentistry, Faculty of Dentistry, Cairo University, Egypt. The procedures were performed after the approval of the Ethics Committee in the Faculty of Dentistry, Cairo University (approval code: 18-4-12) that was in accordance with the Declaration of Helsinki. A protocol was registered in the *ClinicalTrials.gov* (Protocol Registration and Results System) database under ID (NCT03478150), on the 23rd of March, 2018. The study adheres to CONSORT guidelines. The research team bore ultimate responsibility for all the activities associated with the conduct of the research project, including the recruitment of patients and explaining the procedures to the selected ones before carrying out the trial. The procedures and aim of the study were clearly explained to the participants, and then, informed consent was signed by each one of them.

### Sample size calculation

Based on a previous study by Mohan et al. [17], the difference in bacterial counts between at least two groups is 83.3 ± 70. Using power 80% and 5% significance level we needed to study 12 in each group. The number was increased again to a total sample size of 15 in each group to allow for losses of around 25%. Sample size calculation was achieved using PS: Power and Sample Size Calculation software version 3.1.2 (Vanderbilt University, Nashville, Tennessee, USA).

### Study settings and participants

A total of 30 patients were assigned to this study. Patients were randomly divided into two groups, with 15 patients each, according to the type of intervention (A), where (A_1_) represented the group treated with a laser diode, while (A_2_) represented the group treated with ZnO NPs.

### Eligibility criteria of participants

- **Inclusion criteria:**
- Participants aged from 18 to 40.
- Medically free individuals.
- Individuals who haven’t received antibiotic therapy for at least 6 months before sampling.
- Patients with deep cervical carious lesions, particularly in the upper or lower anterior teeth.
- Both male and female patients were included.
- Cooperative patients who approved the trial with all its procedures.

- **Exclusion criteria:**
- Pregnant and lactating mothers.
- Patients with any systemic diseases, mental, or physical disability.
- Patients with Xerostomia.
- Uncooperative patients.

### Selection of teeth

- **Inclusion criteria of teeth**
- Permanent anterior teeth.
- Class V deep dentin caries.
- Absence of spontaneous pain, and negative sensitivity to percussion in the selected teeth.
- Normal response to thermal sensitivity test using hot gutta percha (brief sharp pain).
- Absence of periapical lesions (confirmed through radiographic examination).

- **Exclusion criteria of teeth**
- Teeth with proximal caries lesions
- Teeth with symptoms of irreversible pulpitis or necrotic pulp or with shallow or enamel caries.
- Mobile or periodontally affected teeth.
- Teeth that are tender to percussion.

The antibacterial effect of both laser diode and zinc oxide nanoparticles was evaluated using CFU counted before and after cavity disinfection. Simple randomization was done according to a checklist including the number of participants divided into two groups labeled A_1_ and A_2_. Randomization was generated using the website (www.randomization.com). Allocation of both interventions to each group was done by printing a number corresponding to each treatment group on a piece of paper, which was folded and kept in a dark container. A paper was selected from the container by a person other than the operator, and the antibacterial agent indicated was performed. The operator was blinded until randomization into groups, to avoid biases regarding the application of the antibacterial agents. Also, the microbiologist who carried out the microbiological analysis of dentin samples was blinded to the type of agent applied during treatment. Finally, the treatment results were also blindly assessed by a statistician to avoid the risk of bias of the results in favor of one intervention over the other. Patients were thoroughly screened until the desired population was selected. They were then subjected to full dental examination and diagnosis using dental charts. Once the potentially eligible patients for this study were identified, they were contacted by the researcher who explained the study and ascertained the patient’s interest. Patients who showed interest received more detailed evaluations and preparations, such as meticulous cleaning using an ultrasonic scaler to remove any calculus, followed by polishing of teeth.

A safety monitoring plan was in place for this trial. All patients were observed clinically during and after the interventions for adverse events such as pain, pulp exposure, or soft tissue irritation. Any unexpected events were to be documented and managed according to standard clinical protocols. No adverse events were recorded in either group throughout the study. Given that both interventions (ZnO NPs suspension and 976 nm diode laser) have documented safety in prior dental applications, a formal data safety monitoring board (DSMB) was not convened. However, patient safety oversight was maintained by the principal investigator and attending clinicians, and the trial protocol received prior approval from the institutional ethics committee.

### Partial caries excavation

After application of local anesthesia (4% articaine 1:100,000 epinephrine, Septodont, France), and proper rubber dam isolation, partial removal of carious tissues was performed using a sterile, sharp excavator. According to the guidelines published by the International Caries Consensus Collaboration, caries was selectively removed using a sterile, sharp spoon excavator (Carl Martin GmbH, Solingen, Germany) from all the lateral walls of the cavities, as well, as the superficial carious dentin. Centrally towards the pulp, soft carious dentin was removed until a firm, leathery, slightly moist dentin layer which is still cuttable by excavators was left close to the pulp to avoid its exposure [18–20].

### Obtaining baseline dentin samples (control samples)

A scoop of carious dentin sufficiently large to cover the surface of the blade was taken from the superficial soft carious dentin in the cavity floor by a sterilized sharp spoon excavator other than the one used before in the removal of the carious tissues [21]. The obtained sample was immediately preserved in saline contained in a sterile, disposable test tube, then kept in an ice box to be taken to the laboratory at the Department of Microbiology, Faculty of Medicine, Cairo University, within 2 h [9].

### Application of interventions

#### Disinfection of cavities by laser diode

Each cavity was irradiated in contact mode with a continuous wave of radiation. The laser light was delivered using a diode laser device (Woodpecker LX16 Plus, Guilin Woodpecker Medical Instrument Co., Ltd., China) (Fig. 1), through a flexible 400 µm fiber optic tip attached to a special handpiece. The fiber tip was disinfected prior to each use with 70% ethyl alcohol to prevent microbial cross-contamination, and then inserted into the cavity, positioned perpendicular to the cavity floor. Irradiation with an air cooling system was applied in a circular motion, first clockwise and then counterclockwise, to ensure uniform distribution of the laser beam and to minimise localized heat accumulation at the cavity surface. A total of five irradiation cycles were performed, each lasting 15 s, with a 15-s interval between cycles, in accordance with the manufacturer’s recommendations.

**Fig. 2:**
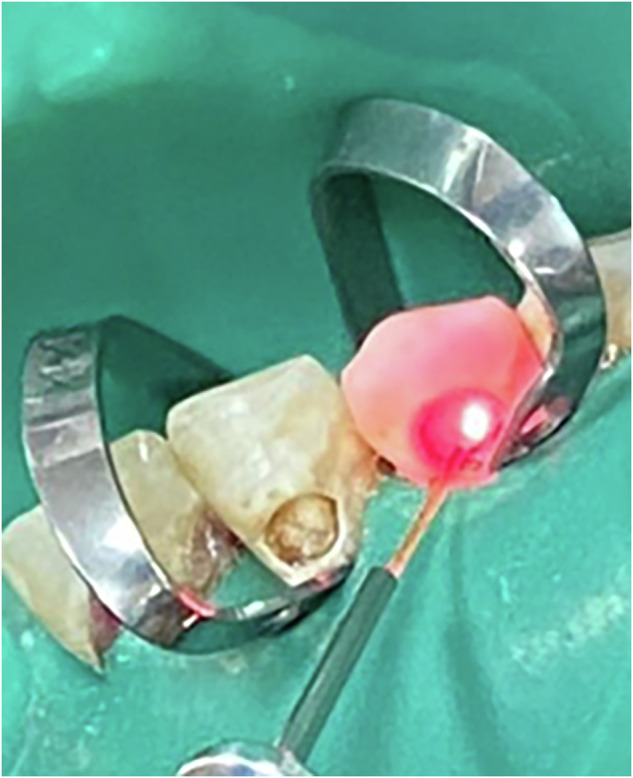
Disinfection of cavity by laser diode. The laser beam is delivered through a 400 μm fibre optic flexible tip. The laser is applied perpendicular to the cavity floor, at a circular motionfirst clockwise, then counter clockwise. Irradiation was done through a total of five cycles, each for 15 s.

The device was operated at a wavelength of 976 nm and an output power of 1.3 W. With a 400 µm tip diameter (0.00126 cm² area), this corresponded to a power density of approximately 1031 W/cm². The energy delivered was 19.5 J per cycle (15 s), resulting in a total of 97.5 J after 5 cycles. The calculated energy density (fluence) was approximately 15,476 J/cm² per cycle, or 77,380 J/cm² in total [9, 22] (Fig. 2) (Table 1).

**Table 1:** Parameters of both interventions.

Parameter	ZnO NPs Group	Diode Laser Group
Agent/device	ZnO nanoparticles in absolute ethanol	Diode laser (Woodpecker LX16 Plus, China)
Concentration/wavelength	50 µg/mL ZnO NPs	976 nm
Preparation	1 mg ZnO NPs in 20 mL ethanol; sonicated	Manufacturer-specified setup
Mode of application	Applied with micro-brush, air-dried, no rinsing	Contact mode, continuous wave, 400 µm fiber tip
Output power/density	Not applicable	1.3 W (~1034 W/cm²)
Cycles/exposure time	Single application (3 min)	5 cycles × 15 s with 15 s intervals
Surface handling post-application	Air-dry; proceed to adhesive	Cooling intervals prevent overheating; proceed to adhesive
Bonding protocol	Immediate adhesive placement and composite restoration	

### Thermal safety and pulp protection

Recent in vitro investigations confirm that 976–980 nm diode lasers operated at 1–1.5 W in continuous wave mode with 400 µm tips do not exceed this threshold when used with intermittent cycles [23]. Direct intrapulpal temperature monitoring was not feasible in the current clinical trial; however, the irradiation protocol was designed to remain within thermally safe limits, supported by recent in vitro and modeling evidence [23]. For further safety, the clinical protocol in the present trial incorporated additional protective measures such as:

- The fiber tip was moved continuously (clockwise/anticlockwise) to avoid localized heating,
- Short irradiation cycles (5 × 15 s) with 15 s cooling intervals were used,
- The applied output (1.3 W, 976 nm) was within the manufacturer’s recommended range for cavity disinfection.

### Disinfection of cavities by zinc oxide nanoparticles

#### Determining the time taken by ZnO NPs to initiate antibacterial activity

To determine the time required for ZnO NPs to initiate their antibacterial activity, the zinc nano-powder was tested before use in the trial. 50 ml of the nanoparticle powder was obtained after ethyl alcohol evaporation and mixed with 50 ml of bacterial suspension of *S. mutans* (5 × 10^3^ colonies). The mix was then cultured on blood agar after 0 (control), 1, 3, 5, and 15 min, by dispensation and spreading of the mixture on agar via a sterile glass rod to ensure a homogenous growth of bacteria, then incubated overnight at 37 °C. Finally, the remaining bacterial colonies were counted [24]. The main purpose of this preclinical test was to determine the reasonable time interval that is sufficient for the ZnO NPs to initiate their antibacterial effect, and at the same time, convenient for clinical application without consuming time or causing patient discomfort.

### Application of zinc oxide nanoparticles to the cavities

After obtaining the baseline sample, the cavities were washed using sterile saline and dried with sterile cotton swabs. The ZnO NPs in ethanol solution were applied using a sterilemicro-brush (3 M, St Paul, MN, USA) soaked in the suspension, then a gentle air stream was applied to the cavity for 30 s without rinsing to maximize nanoparticles adherence and ensure full vaporization of ethanol without excessive dryness of dentin, leaving the ZnO NPs to infiltrate into the dentinal tubules [14, 25]. ZnO NPs were left for 3 min in the cavity before taking the second sample to allow for sufficient time for the zinc nanoparticles to begin producing their antibacterial effect [24].

### Post-intervention dentin samples

After the application of interventions, a second dentin scoop was obtained using a different sterile spoon excavator. As previously done with baseline (control) samples, post-treatment samples were preserved in sterile tubes containing saline, kept in an icebox, and then transferred to the laboratory within 2 h [9].

### Restorative procedures

**Fig. 3:**
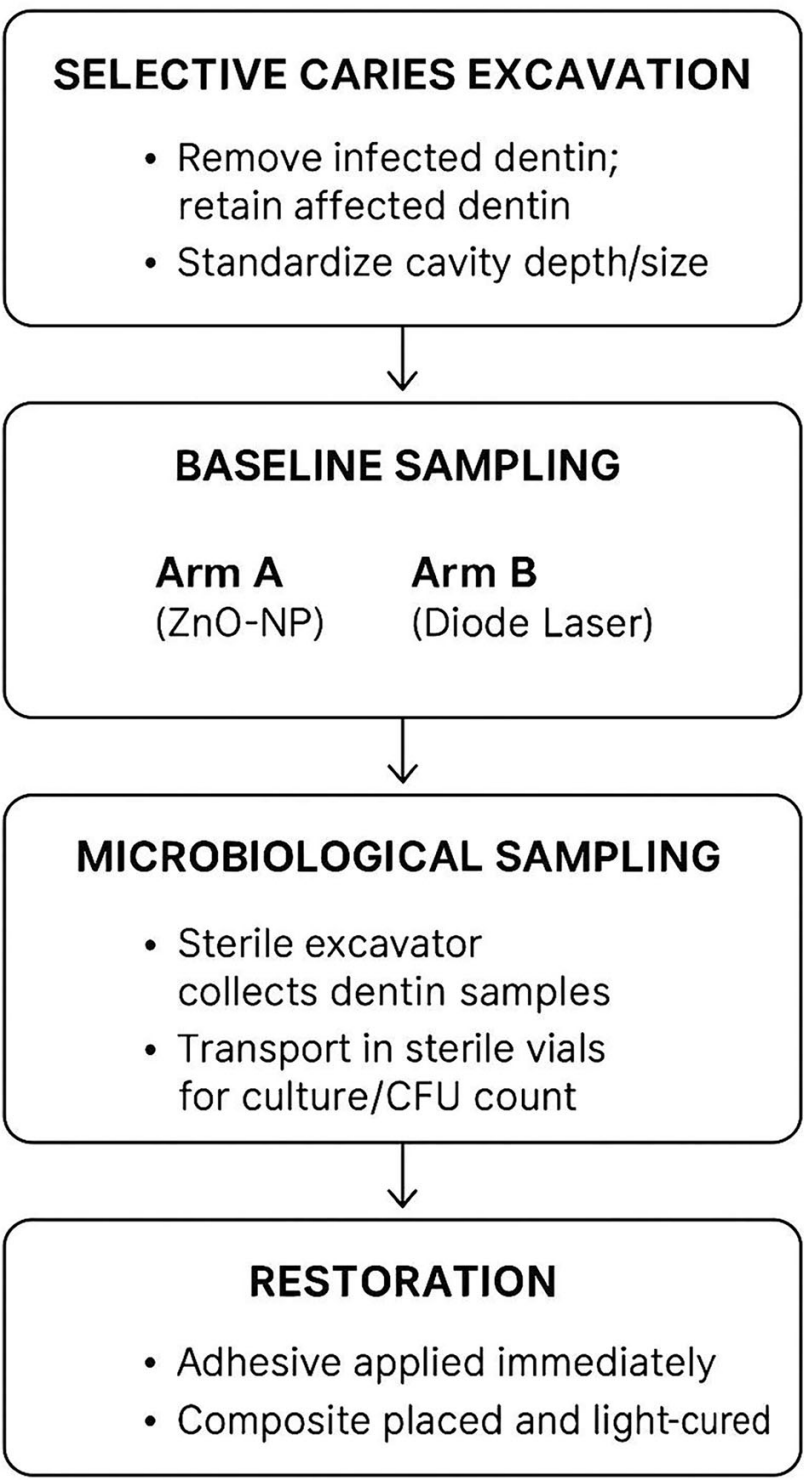
Clinical workflow. Caries were selectively removed from all the cavity walls, then a baseline dentine sample was taken. Application of interventions was done, the a post-intervantion sample was taken. The cavities were restored using resin composite, finished and poilshed, while the samples were taken to the laboratory for microbiological analysis.

The enamel walls and cavity outlines were prepared and finished. Afterwards, bonding procedures were done, first, by selective etching of the enamel walls with 37% phosphoric acid gel, for 20 s [26]. Then, the etching gel was thoroughly rinsed with a copious water stream and dried with a gentle air stream until the etched enamel surfaces turned chalky white. Finally, the adhesive bonding agent (Bisco® All Bond Universal) was applied to the whole cavity and agitated by a saturated bond brush for 20 s. The bond layer was air-thinned with compressed air until a glossy, fixed film layer was obtained and cured for 20 s according to the manufacturer’s recommendation by an LED-curing unit (Demi Ultra, Kerr Corp., Orange, CA, USA). The restorative nano-filled resin composite material Filtek TM Z350 XT (3MTMESPETM, St. Paul, MN, USA) was incrementally packed and adapted to the cavity walls, then cured for 30 s [27]. The final contouring, finishing, and polishing of the restoration were done with the aid of diamond finishing stones (Komet GmbH, Lemgo, Germany), in addition to the flexible disks (Sof-Lex, 3 M ESPE, St. Paul, MN, USA), followed by rubber points (Kerr Corp., Orange, CA, USA) to ensure a smooth, lustrous surface Fig. 3.

### Determination of the antibacterial effect of the interventions

From each treated cavity, two dentin samples were obtained, one before and one after treatment with the antibacterial. Therefore, a total of 60 test tubes (samples) were transferred to the laboratory for analysis. First, the test tubes containing the dentin samples were shaken in a vortex for 30 s to scatter bacterial aggregates, then decimal dilutions were prepared in sterile saline (0.9% NaCl). 50 μl of each bacterial dilution was spread on the surfaces of Mitis Salivarius agar (HI Media Laboratories Pvt. Ltd., India) plates via a sterile glass rod to obtain a homogenous growth of bacteria. The agar plates were then incubated in a 5% CO_2_ environment inside a candle jar, for 48 h. Following the incubation, the bacterial count of *S. mutans* (CFU) was determined in samples taken before and after treatment with the interventions. The microbiological assessment was performed by a microbiologist who was blinded to the type of treatment the specimens received to avoid the risk of bias [9, 28, 29].

### Statistical analysis

The mean and standard deviation values were calculated for each group in each test. Data were explored for normality using Kolmogorov-Smirnov and Shapiro-Wilk tests, microbiological data showed parametric (normal) distribution. A paired sample t-test was used to compare two groups in related samples. An Independent sample t-test was used to compare two groups in non-related samples. A two-way ANOVA test was used to test the interactions between different variables. For non-parametric data, Mann-Whitney test was used to compare two groups in non-related samples. The significance level was set at *P* ≤ 0.05. Statistical analysis was performed with IBM® SPSS® Statistics Version 20 for Windows.

## Results

### Characterization of zinc oxide nanoparticles

#### The morphological analysis of zinc oxide nanoparticles

##### Transmission electron microscopy

**Fig. 4:**
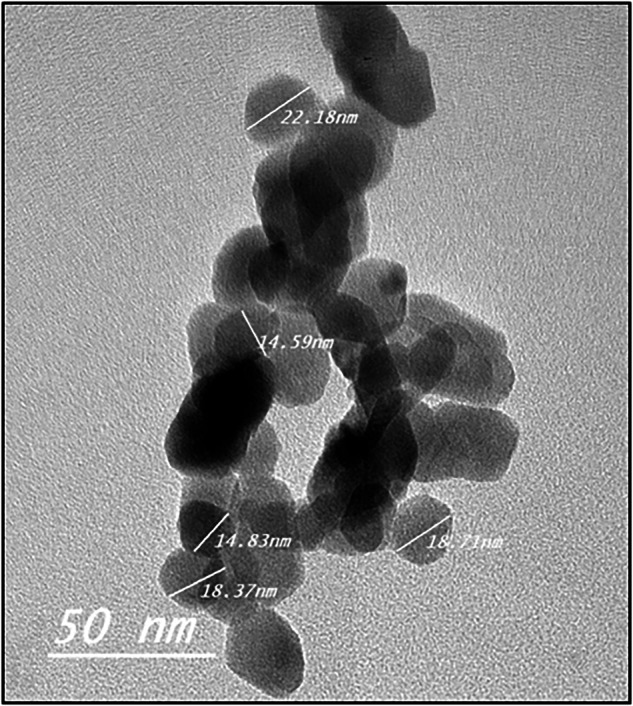
Photomicrograph of the prepared ZnO nanoparticles. The photomicrograph showed spherical to polygonal shaped ZnO nanoparticles with particle size ranging from 14.59 to 22.18 nm.

The resulting photomicrograph showed that the NPs were in the range of 14.59–22.18 nm with an 18 nm average diameter having round to polygonal shape (Fig. 4).

### X-ray diffraction analysis of zinc oxide nanoparticles

**Fig. 5:**
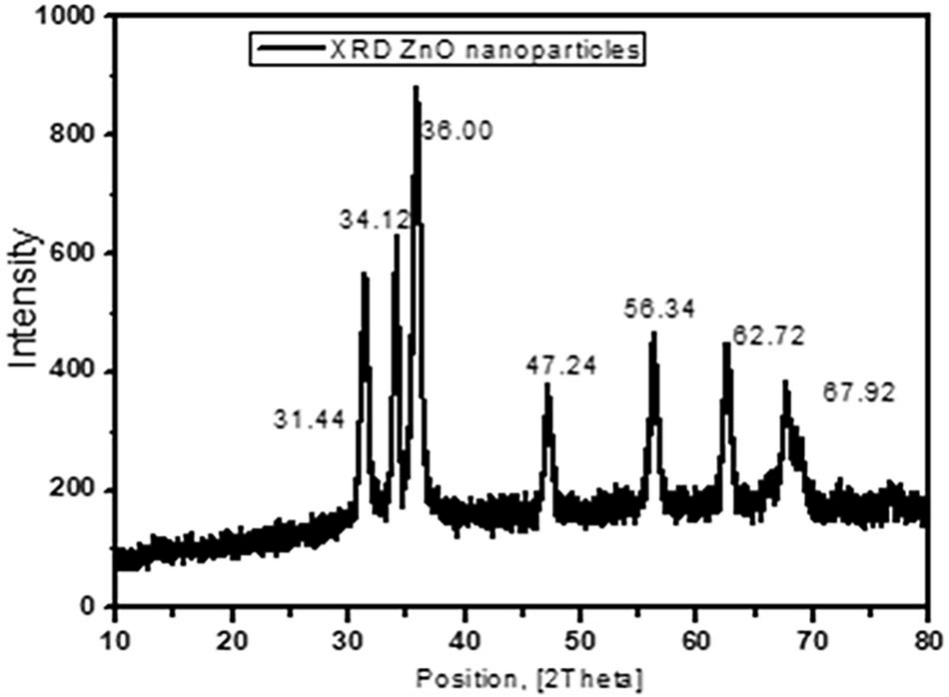
The XRD pattern of the synthesized ZnO NPS. The XRD pattern of the synthesized ZnO NPs showed seven peaks at 2*θ* values of 31.44°, 34.12°, 36°, 47.24°, 56.34°, 67.72°, and 67.92°.

The XRD pattern of the synthesized ZnO NPs showed seven peaks at 2*θ* values of 31.44°, 34.12°, 36°, 47.24°, 56.34°, 67.72°, and 67.92°. The narrow and sharp diffraction peaks indicate that the synthesized ZnO NPs have a highly crystalline structure (Fig. 5).

### Determining the time taken by ZnO NPs to initiate antibacterial activity

**Fig. 6:**
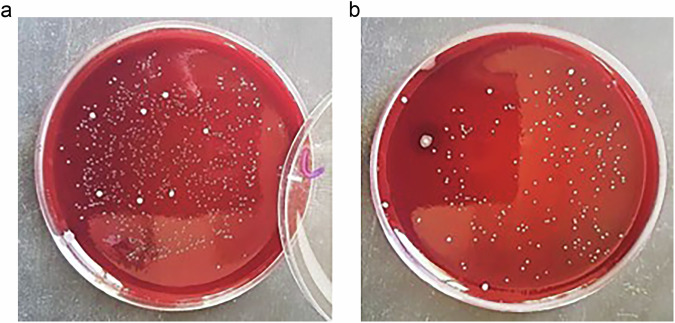
Determination of the time taken by zinc oxide nanoparticles to produce its antibacterial effect against S. mutans. **a** Bacterial colonies of *S. mutans* on blood agar before intervention (control). *S. mutans* was cultured on blood agar without application of ZnO NPs to determine the initial count of bacterial colonies. **b** Bacterial colonies of *S. mutans* after 3 min exposure to ZnO- NPs. The picture shows a reduced number of bacterial colonies of S mutans after 3 min exposure to ZnO NPs, compared to the initial bacterial count before application of ZnO NPs.

ZnO NPs antibacterial activation time: *S. mutans* CFUs were reduced significantly after 3 min of exposure to ZnO NPs (Fig. 6a, b).

### Colony-forming units/mL (CFU) results

**Fig. 7:**
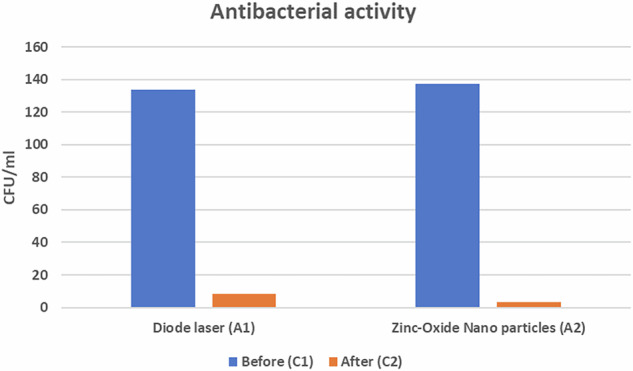
Bar chart representing CFU in each group. Within each group (A1) and (A2), there was a statistically significant difference between baseline (C1) (control) group and post-treatment (C2) group (p 0.001).

Within each group (A1) and (A2), there was a statistically significant difference between baseline (C1) (control) group and post-treatment (C2) group (*p* < 0.001) (Fig. 7). The highest mean value was found among the baseline samples(C1) in both groups, while the lowest mean value was found among the post-treatment samples (C2) in both A1 and A2 (Table 2).

**Table 2:** Mean and standard deviation (SD) values of different groups.

Variables	CFU/ml				
	Diode laser A1		Zinc oxide nanoparticles A2		*P*-value
	Mean	SD	Mean	SD	
Before	133.67	10.60	137.33	22.51	0.573 ns^a^
After	8.27	0.80	3.47	0.74	<0.001
*P*-value	<0.001		<0.001		

^a^Non-significant.

**Fig. 8:**
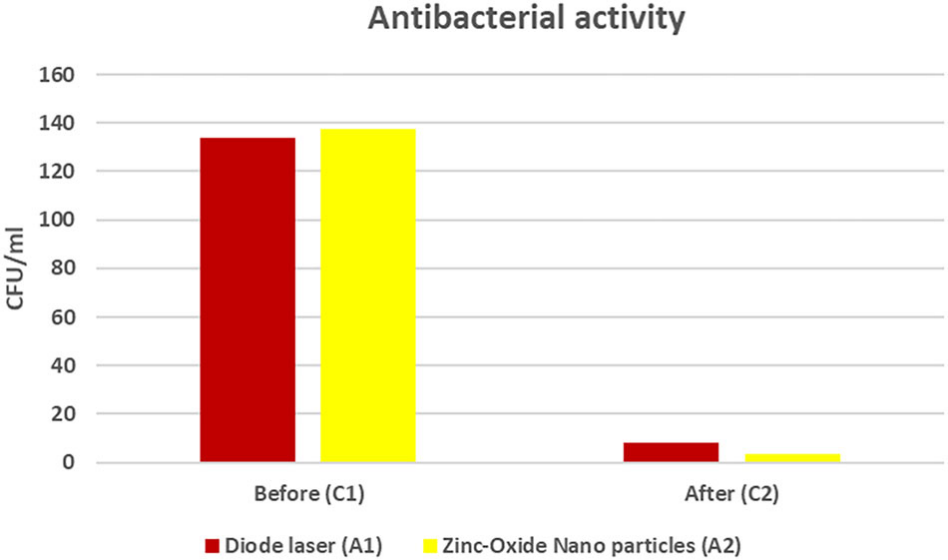
Bar chart representing CFU for different groups. There was a non-statistically significant difference between the two control groups (baseline samples) (p = 0.573). However, after the disinfection of cavities, there was a statistically significant difference between diode laser (A1) and Zn- O NPs (A2) groups (p 0.05).

There was a non-statistically significant difference between the two control groups (baseline samples) (*p* = 0.573). However, after the disinfection of cavities, there was a statistically significant difference between diode laser (A1) and Zn- O NPs (A2) groups (*p* < 0.05) (Fig. 8). The highest mean value was found among the specimens disinfected by diode laser (A1), while the least mean value was found among the specimens treated by ZnO NPs (A2).

**Fig. 9:**
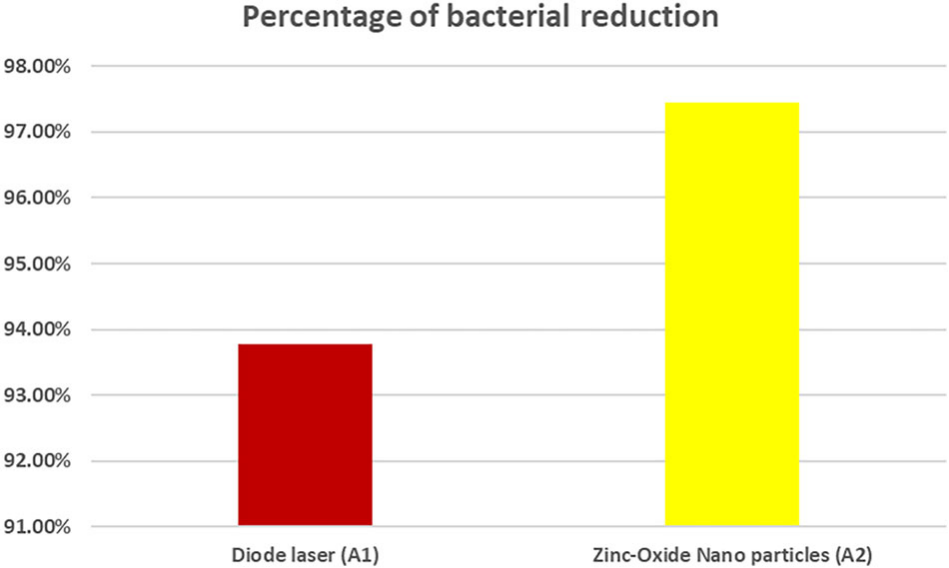
Bar chart representing percentage of bacterial reduction between different groups. The bar chart shows a higher percentage of bacterial reduction in the zinc oxide nanoparticles group compared to the diode laser group.

The microbiological analysis showed a statistically significant difference between diode laser (A1) and ZnO NPs (A2) groups where (*p* < 0.05) (Fig. 9). The lowest mean value of bacterial reduction was found in the diode laser (A1) group, while the highest mean value was found in ZnO NPs (A2) group (Table 3).

**Table 3:** Mean and standard deviation values of the percentage of bacterial reduction.

	Percentage of bacterial reduction	
	Mean%	SD%
Diode laser A1 Zinc oxide nanoparticles A2	93.77% 97.4%	0.81% 0.60%
*p*-value	<0.001	

## Discussion

In the past few years, dental treatment has been shifting towards a more conservative approach, focusing on prevention and early detection of caries, therefore, the term “minimally invasive dentistry” has evolved. As a result, preventive and conservative procedures have become of great importance, as they can greatly enhance the oral health of patients in the long run [5, 30].

Management of caries involves the removal of carious and necrotic tooth structure, with subsequent restoration of the remaining dental tissues [4]. However, the success of treatment becomes more challenging due to the presence of residual bacteria such as *Streptococcus mutans* in the smear layer. The remaining bacteria under restorations may stay viable for a long time and are capable of invading the dentinal tubules up to a depth of 1 mm [31, 32]. Unfortunately, caries removal and sealing of the carious lesion are not able to eliminate the residual cariogenic microorganisms [33–37]. Accordingly, the role of cavity disinfecting agents becomes critical to get rid of the remaining intra-tubular bacteria and protect the pulp vitality, especially in deep caries [4, 32].

As an alternative to conventional complete caries removal, selective caries excavation is considered a reasonable approach to minimize the risk of pulp exposure and preserve its vitality. This technique allows a change in the microenvironment of carious lesions, decreasing the diversity and number of cariogenic bacteria, and consequently, arresting the progression of caries. Incomplete caries excavation involves partial removal of carious dentin, however, the exact amount of carious tissues to be removed is still controversial [38]. Some studies recommended the removal of carious dentin until firm dentin remains at the periphery of the cavity, while centrally, carious dentin that has different textures (soft, firm, or leathery) and is still cuttable by excavators, is left overlying the pulp [19, 20]. The remaining carious tissue is then carefully sealed with a permanent restoration, arresting caries progression and permitting the pulp-dentine complex to produce its reparative reactions [39].

A variety of adjuncts have been used over the past few years for inhibiting the growth of residual bacteria in deep caries. Chemical disinfecting agents have shown a promising antibacterial effect, however, their action is still limited by some side effects, such as their interaction with the restorative material, or causing alterations in the structure of dental tissues. As a result, other antibacterial alternatives are required to overcome such problems. Laser diode and Zinc oxide nanoparticles are among the recent advances used in cavity disinfection [8].

In the current study, the diode laser was compared to the use of zinc oxide nanoparticles as a way of carious dentin disinfection. Diode is a category of lasers ranging from 810 to 1064 nm, within the infrared spectrum of light. This type of laser is among the most commonly used lasers in dental applications, owing to their relatively compact size, lightweight, ease of application, and economic price [40, 41]. In particular, diode lasers in the range of 976–980 nm are increasingly available in dental practice. Entry-level units such as the Woodpecker LX16 (used in the current study) are relatively cost-effective compared to erbium lasers, and their operation requires only short training sessions for safe handling. Previous studies [42] confirm their growing clinical acceptance for disinfection and soft tissue management. Although 810 and 940 nm diode lasers are also used in dentistry, the 976 nm wavelength offers higher absorption in water and hydroxyapatite, making it particularly suitable for hard-tissue disinfection. This wavelength has been shown to produce superior bacterial reduction on dentin surfaces compared with lower wavelengths, while remaining within the thermal safety threshold when applied at ≤1.5 W in continuous mode [23]. The protocol used in this trial (5 cycles × 15 s irradiation with intervals) adds approximately 2–3 min to the procedure, which is clinically feasible and comparable to other disinfection adjuncts (e.g., chlorhexidine rinses, photoactivated disinfection).

The antibacterial action of laser can be referred to its photothermal and photo-disruptive effects [40, 43]. Photothermal effects of laser occur when target tissues absorb the light energy from laser, which is then converted into thermal energy, causing several effects like tissue ablation, photocoagulation, or photopyrosis [31]. Regarding its photo-disruptive effect, laser light can cause the breakdown of microbial structures [44]. More importantly, diode laser has been proven to display remarkable antimicrobial activity without affecting the host cells [9].

ZnO NPs have superior antibacterial, antifungal, electrical, chemical, and optical properties, which make them cytotoxic against bacterial cells at small concentrations, without adversely affecting human cells [45–49]. Another critical factor that makes zinc oxide nanoparticles stand out among other metal nanoparticles is their relatively low cytotoxicity. ZnO NPs are trace elements that are normally present in the human body, which makes them biocompatible with human cells [14]. At present, ZnO nanoparticles are not approved as a stand-alone intraoral medicament by regulatory agencies such as the FDA or EMA. However, ZnO is a GRAS-listed (Generally Recognized as Safe) material and is widely used in dental cements, pastes, and restorative materials. Several recent preclinical and translational studies [50, 51] have demonstrated its safety and antibacterial efficacy in oral applications, suggesting strong potential for future regulatory approval pending toxicity and biocompatibility data. While diode lasers are already clinically accessible, ZnO NPs suspensions remain at the investigational stage. Therefore, their near-term role may be as an adjunctive, chairside-prepared disinfectant in research or specialized settings, with future translation dependent on regulatory pathways and formulation standardization.

Based on this data, the present clinical trial was primarily conducted to investigate the antibacterial activity of laser diode and ZnO NPs against cariogenic bacteria; particularly *S. mutans*, when used as cavity disinfectants. The antibacterial effect of both interventions was tested via CFU. The microbiological results showed a statistically significant difference between the dentin samples, before and after cavity disinfection, within each group, whereas the dentin samples after cavity disinfection showed a significant reduction in the bacterial count. This can be attributed to the antimicrobial potential of both, diode laser and ZnO NPs. Comparing the two groups, the results showed a statistically significant difference between the laser diode and the ZnO NPs groups, where the ZnO NPs group demonstrated a higher reduction in the bacterial count of *S. mutans* compared to the laser diode group_._

On the other hand, there was not a statistically significant difference between the baseline samples of both groups; laser diode and ZnO NPs. Within each group, the highest number of bacterial colonies was found among the baseline samples, while the lowest number of colonies was found in the samples taken after disinfection. Comparing the post-treatment samples in both groups, the highest number of bacterial colonies was found in the group treated with a laser diode, whereas the lowest bacterial count was found in the other group, which was treated with ZnO NPs. This gives a clear indication that disinfection using ZnO NPs was more effective than laser diode, owing to the strong antibacterial properties of zinc oxide nanoparticles. So, under the limitations of this study, the proposed hypothesis suggesting that there will be a difference in the bacterial count of *S. mutans* after application of laser diode and ZnO NPs as cavity disinfectants was accepted and supported by the results of this clinical trial.

The significant antibacterial activity of ZnO NPs shown in the results can be referred to several mechanisms. Zinc nanoparticles are capable of generating oxygen compounds (H_2_O_2_), which produce reactive oxygen species (ROS). ROS is harmful to living bacterial cells. Literature has shown that bacterial inhibition increases with increasing concentration of ZnO NPs and consequently, increasing the production of H_2_O_2_, so the antibacterial effect, in turn, increases. As a result, the bacterial cell membranes are damaged, causing a defect in the components of the cell, and eventually, cell death occurs. It has also been reported that the antibacterial activity of ZnO NPs is related to their small size, being 250 times smaller than a bacterial cell, which facilitates their binding to the bacterial cell wall, causing cell destruction and death [48]. Another suggested mechanism for the antibacterial activity of ZnO NPs is the release of Zn^+2^ cations that can degrade cell membranes and then interact with the intracellular components. This interaction between zinc ions and the bacterial cell wall is mainly due to the electrostatic forces produced by their opposite charges. However, further investigations are needed to determine the exact mechanism of the antibacterial activity of ZnO NPs.

### Limitations

The sample size is relatively small, so the results may not be generalizable to a broader population.

A limited demographic (e.g., specific age groups, regions, or dental health conditions) may also affect external validity. Additionally, Variability in Clinical Conditions, such as differences in caries severity, patient oral hygiene, and dietary habits, could introduce variability in outcomes. Other challenges including microbial recurrence and resistance to zinc oxide nanoparticles, as well as difficulty in standardizing treatment conditions (Saliva composition, oral pH, and individual patient variations) may affect antibacterial efficacy. Also, since bacterial reduction was assessed via culture techniques, variations in sample collection and handling might have introduced unintentional bias.

Another setback is that the potential effect of ZnO-NP cavity disinfection on dentin bond strength was not measured in the present trial; future work, including micro-tensile bond strength and interfacial microscopy, is warranted to confirm adhesive compatibility.

Ensuring proper blinding might have been difficult, especially operator non-blinding, potentially introducing bias. Therefore, authors recommend future multicenter clinical trials with long-term follow-up and more strict blinding strategies to strengthen evidence on long-term clinical outcomes.

## Conclusions

The results of this clinical trial demonstrate that cavity disinfection with spherical zinc oxide nanoparticles approximately 25 nm in size, and at a concentration of 50 µg/mL in ethanol-based solution exhibits effective antibacterial activity against *Streptococcus mutans*. This activity was stronger than that of diode laser operating at an output power of 1.3 W, and a wavelength of 976 nm.

## Supplementary information

CONSORT checklist

## Data Availability

The dataset used and the analyzed data are available from the corresponding author upon reasonable request.
